# The Pattern of Malignancies in Down Syndrome and Its Potential Context With the Immune System

**DOI:** 10.3389/fimmu.2018.03058

**Published:** 2018-12-19

**Authors:** Daniel Satgé, Markus G. Seidel

**Affiliations:** ^1^Laboratoire Biostatistiques Epidémiologie Santé Publique, Team Cancer (EA 2415), and Oncodefi, Institut Universitaire de Recherche Clinique, Montpellier, France; ^2^Institut Universitaire de Recherche Clinique, Biostatistics, Epidemiology and Public Health EA2415, Montpellier, France; ^3^Division of Pediatric Hematology Oncology, Department of Pediatric and Adolescent Medicine, Medical University Hospital, Graz, Austria

**Keywords:** down syndrome, immune surveillance, immune defect, trisomy 21, cancer, cancer incidence, tumor profile, cancer protection

## Abstract

The immune surveillance theory of cancer posits that the body's immune system detects and destroys randomly occurring malignant cells. This theory is based on the observation of the increased frequency of malignancies in primary and secondary immunodeficiencies, and is supported by the successful demonstration of immune augmentation in current oncological immune therapy approaches. We review this model in the context of Down syndrome (DS), a condition with a unique tumor profile and various immune defects. Children and adults with DS are more prone to infections due to anatomical reasons and a varying degree of T- and B-cell maturation defects, NK cell dysfunction, and chemotactic or phagocytic abnormalities. However, despite an increased incidence of lymphoblastic and myeloblastic leukemia of infants and children with DS, individuals with DS have a globally decreased incidence of solid tumors as compared to age-adjusted non-DS controls. Additionally, cancers that have been considered “proof of immune therapy principles,” such as renal carcinoma, small cell lung carcinoma, and malignant melanoma, are less frequent in adults with DS compared to the general population. Thus, despite the combination of an increased risk of leukemia with detectable immune biological abnormalities and a clinical immunodeficiency, people with DS appear to be protected against many cancers. This observation does not support the immune surveillance theory in the context of DS and indicates a potential tumor-suppressive role for trisomy 21 in non-hematological malignancies.

## Introduction

According to the cancer immune surveillance theory, the immune system detects and destroys cancer cells that develop randomly in various tissues (1–3). In line with this model, medical conditions with inherited or acquired immune deficiency should also be associated with an excess of all types of cancers: malignant cells would escape surveillance by an impaired immune system and therefore proliferate (4). However, recent reviews of cancer events in individuals with primary immune deficiencies show only a mildly increased frequency of cancers and a particular distribution of cancer types, questioning this model (5, 6).

Here, we review the immune surveillance model in the context of trisomy 21, or Down syndrome (DS), a condition that is extensively studied for its immune defects (7) and unique tumor profile (8). Data on this well-defined genetic condition do not fit with the cancer immune surveillance theory because, despite decreased immune efficiency, people with DS have a reduced incidence of solid malignancies. This conflict raises important questions and offers new avenues to understand the poorly explored topic of natural protection against cancer.

## Cancers and Immune Function in DS

### Unique Cancer Distribution in DS

DS, due to a supernumerary chromosome 21, is the most frequent viable chromosome anomaly, with an occurrence of 1 in 700–1,000 live births worldwide. Currently, the life expectancy of people with DS is >50 years (9), permitting evaluation of the occurrence of frequent adult cancers. Although DS was formerly suspected to increase the general cancer risk because of an increased leukemia incidence in childhood, age-adjusted epidemiological studies have established that individuals with DS have a decreased global malignancy burden (10, 11). This is mainly due to reduced frequency of adult solid tumors that account for nearly half of the tumor burden in the general adult population (10–12), but also due to a reduced incidence of many solid tumor types of childhood (13).

Additionally, cancer distribution in DS differs from that in the general population (8). For instance, breast cancer and neural malignancies, such as neuroblastoma (14) and medulloblastoma (15), have a decreased incidence in DS. However, some cancers, especially early childhood leukemia and testicular germ cell tumors in young men (16), only in part attributable to cryptorchidism and testicular microlithiasis, and, to a lesser extent, cancers of the liver and stomach, also appear to be more frequent in individuals with DS than in the general population (10–12) (Table 1).

**Table 1:** Cancer distribution in Down syndrome.

**Increased frequency**	**SIR**	**Observed/expected**	**References**
**Children**			
Acute myeloid leukemia	11.8 (7.11–18.5)		(11)
Acute lymphoid leukemia	13.0 (8.74–18.5)		(11)
Germ cell tumors		5%/1.1%	(13)
**Adults**			
Testicular cancer	4.8 (1.8–10.4)		(10)
Gastric carcinoma	1.65 (0.33–4.83)		(11)
	1.5 (0.3–4.5)		(10)
Liver carcinoma	1.19 (0.02–6.65)		(11)
	2.4 (0.1–13.2)		(10)
**DECREASED FREQUENCY**			
**Children**			
Neuroblastoma and PNETs		0/5.40 (*p* = 0.005)	(14)
Medulloblastoma		1/7.11 (*p* = 0.007)	(15)
**Adults**			
Breast carcinoma	0.16 (0.03–0.47)		(11)
Lung carcinoma	0.10 (0.00–0.56)		(11)
Prostate carcinoma	0.0 (0.0–0.03)		(11)
Colon carcinoma	0.37 (0.04–1.34)		(11)
ENT and oral carcinoma	0.00 (0.00–1.15)		(11)
Malignant melanoma	0.25 (0.03–0.89)		(11)

*SIR, Standardized Incidence Ratio; PNETs, Primitive neuroectodermal tumors; ENT, Ear Nose and Throat; Ref, references*.

### Impaired Immune Function in DS

DS is the most common recognizable genetic syndrome associated with immune defects (7), which are detectable as early as fetal development (17). Abnormal parameters of the immune system were identified following evidence of frequent respiratory infections responsible for recurrent hospitalizations and frequent otitis media (7, 18, 19). Overall, the risk to die from an infection is 12-fold higher in patients with DS as compared to individuals without DS (20). DS-related immune impairment is complex and varies among individuals, affecting mainly B cells and humoral (including mucosal) immunity, T-cell-mediated immunity, NK cells, and neutrophils (21–26) (Table 2). Some features are reminiscent of premature immune senescence (23) and common variable immune deficiency (23–26), leading to immune dysregulation with relative imbalance between pro-inflammatory and anti-inflammatory immune responses. In line with this, people with DS are more prone to autoimmune diseases of the thyroid (Graves disease, Hashimoto thyroiditis), pancreas (type 1 diabetes mellitus), gut (celiac disease), and skin (alopecia areata, vitiligo). These autoimmune manifestations usually appear earlier in life and are more frequently associated in comparison to persons without DS (18).

**Table 2:** Sum of reported immune abnormalities and other factors that potentially contribute to an increased risk of infections in Down syndrome.

**Compartment**	References^*^
**T CELLS**	
Normal or mildly-moderately decreased T cell numbers	(21)
Reduced proportion of naïve T cells	(22)
Increased proportion of T cell receptor γδ+ T cells	(22)
Impaired T cell maturation and memory development	18
Normal or decreased mitogen stimulation response (SEB, PHA)	(22)
Impaired functional activity of T regulatory cells	(21)
**B CELLS AND HUMORAL IMMUNITY**	
Mild–moderate decrease in B cell numbers	(23)
Normal transitional but reduced naïve, effector, and memory B cells	(23)
Activation and adherence defect	(21)
Lower serum levels of IgM, higher serum levels of IgA and IgG; inconsistent reduction of IgG2, reduction of IgA in saliva	(21, 23)
Impaired molecular maturation of IgA and IgM	(23)
Impaired specific antibody production against protein antigens	(24)
Impaired specific antibody production against polysaccharide antigens	(24)
**NK CELLS AND INNATE IMMUNITY**	
Reduced functionality of NK cells	(25)
**PHAGOCYTE NUMBER AND/OR FUNCTION**	
Impaired neutrophil chemotaxis and, inconsistently, of phagocytosis	(26)
**Non-immunological factors**	
Anatomical: laryngo- and/or tracheomalacia, macroglossia, ear
abnormalities; obstructive sleep apnea	(27)
Gastro-esophageal reflux and aspiration	(27)

* *According to (18, 21, 24, 25, 25, 27) in part reviewed and summarized by Ram and Chinen (7) and Kusters et al. (22); SEB, staphylococcal enterotoxin B; PHA, phytohemagglutinin A*.

At least four genes mapping to chromosome 21 are involved in immune functions and have been postulated to account for some of the biological and clinical findings related to immunity in DS: interferon alpha receptor 1 (*IFNAR1*); interferon gamma receptor chain 2 (*IFNGR2*); ICOS ligand (*ICOSLG*), which encodes CD275; and integrin beta chain 2 (*ITGB2*), which encodes CD18. These four genes should theoretically be overexpressed through a gene dosage effect, since three copies are present in DS cells, including leukocytes. However, only CD18 is significantly elevated in individuals with DS (19). Additionally, two other genes on chromosome 21, DS critical region 1 (*DSCR1*) and dual-specificity tyrosine phosphorylation-regulated kinase 1A (*DYRK1A*), are involved in a regulatory circuit that includes nuclear factor of activated T-cells (NFAT) proteins, potentially contributing to a modulation of the immune response (18, 19).

## No Increased Incidence of Most Solid Cancer Types Despite Increased Risk of Infections and Biological Abnormalities of the Immune System in DS

**Figure 1:**
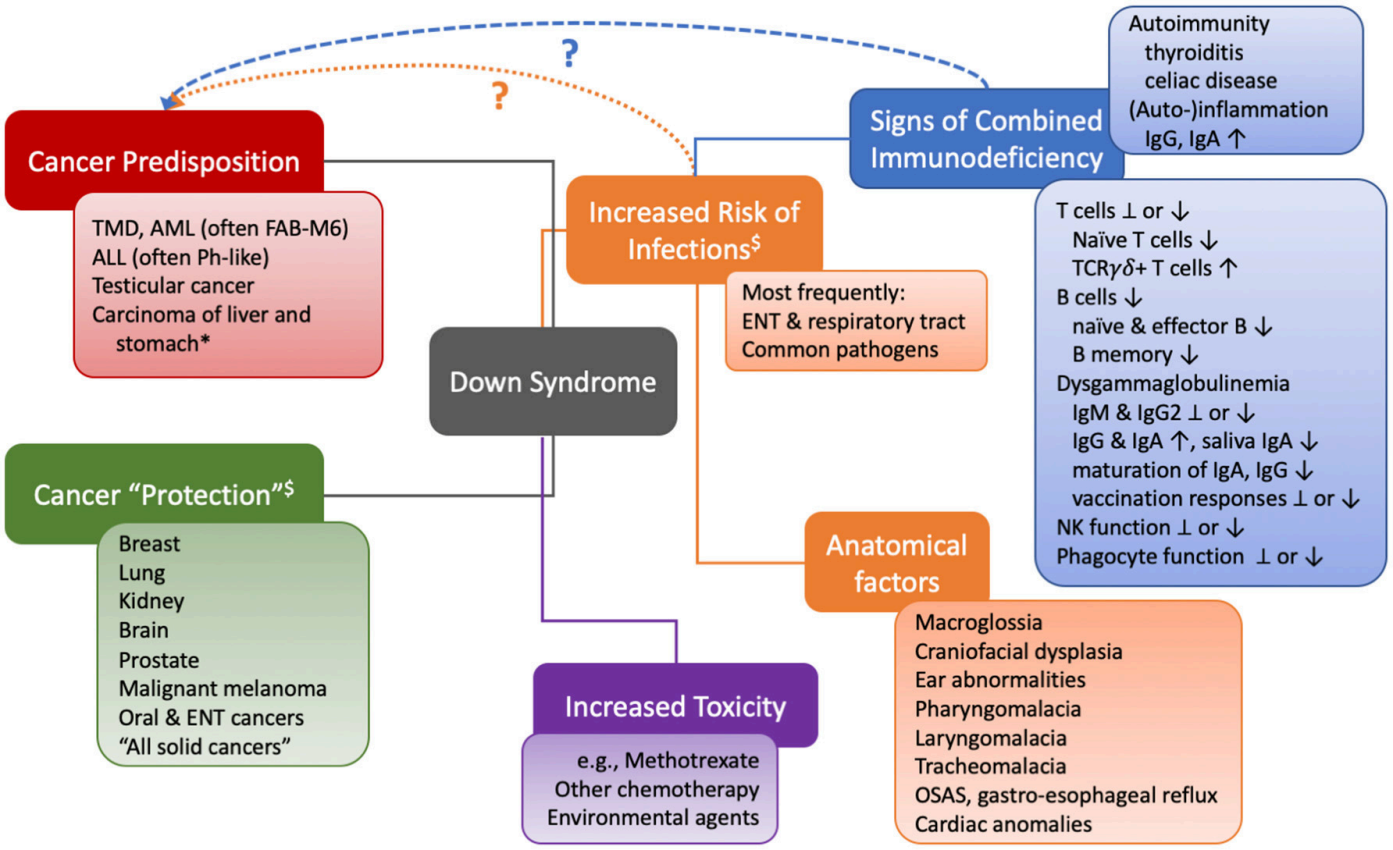
Visual contextualization of cancer risks and the immune system in Down syndrome. *different risk ratios (increased vs. decreased) were detected in different studies. ^$^Caveats: most but not all studies took into account age-matched control cohorts, but not social and environmental factors (smoking, UV, diet, institutionalization, sexual activity…), endocrine differences, aging, or senescence. TMD, transient myeloproliferative disorder; AML, acute myeloid leukemia; FAB-M6, French American British classification M6 (megakaryocytic); ALL, acute lymphoblastic leukemia; Ph-like, Philadelphia chromosome-like signature, often associated with mutations in *IKZF1;* ENT, ear nose throat; TCR, T cell receptor; Ig, immunoglobulin; NK, natural killer cell; OSAS, obstructive sleep apnea syndrome.

Decreased efficiency of immune cells should result in an increased cancer frequency, because escape from impaired immune surveillance would enable cancer cells to survive and proliferate. Individuals with DS have an increased rate of mortality from infections as compared to the general population. This susceptibility, together with a variety of biological abnormalities of the immune system that are reminiscent of common variable or combined immunodeficiency (CVID or CID, respectively), could prompt the assumption that immune surveillance is impaired. Additionally, mucosal immunity may be impaired and fail to control infections of the gut that contribute to carcinogenesis. In fact, the observation of mildly increased mortality from gastric and liver cancers suggests that extrinsic mechanisms of tumorigenesis such as chronic infection or inflammation, in combination with potentially impaired elimination of tumor cells by the immune system, could be at play (Figure 1).

However, individuals with DS have an, age-corrected, decreased frequency of those solid tumor types that comprise half of the total cancer burden of the general population (11), arguing against a role of (globally impaired) immune surveillance. Further, people with DS also have a reduced cancer frequency compared to people with other conditions of intellectual disability, who develop cancers at rates similar to the general population (28, 29). This suggests that the excess of genetic material on the supernumerary chromosome 21 provides protection against certain types of malignancy.

Additionally, other observations fail to support the role of immune surveillance in DS. First, three malignancies that have been considered proof of immune therapy principles—kidney carcinoma, small cell lung carcinoma, and malignant melanoma (5)—are not more frequent in people with DS. On the contrary, kidney cancer, lung cancers including small cell carcinoma, and malignant melanoma have a decreased incidence in DS (11). Second, medulloblastoma, a neural cell embryonal brain malignancy, is rare in children with DS (15). An immune mechanism would hardly explain why, in the same epidemiological study, the frequency of glial malignancies of the brain was not found to be reduced in children with DS (15). Third, nearly 1 out of 20 infants with DS develops a transient myeloproliferative disorder that spontaneously disappears in most affected individuals during the first months of life (30). These spontaneous regressions of premalignant abnormal proliferation occur at a time when the immune system is weak and immature, and it is even more impaired in children with DS.

Even in typically malignancy-prone primary immune deficiencies, an increased risk of leukemia is attributable to an intrinsic mechanism of oncogenesis, in parallel, rather than as a consequence of the immune defect (5). Thus, the increased frequency of leukemia observed in people with DS is unlikely to be due to a lack of immune surveillance. The genetic etiology of myeloid (typically megakaryoblastic) or lymphoblastic (often Philadelphia-like, high-risk) leukemias is complex and beyond the topic of this review. The slightly increased risk of gastric and liver cancers in DS in part reminds of that of patients with predominantly antibody deficiencies such as CVID (31), who also show reduced mucosal immunity, which in turn could facilitate chronic infection, inflammation, and thereby, stochastically, increase the risk of malignant transformation (Figure 1). The inconsistently detected defects in T-cellular immunity appear to play a minor role clinically, as the pattern of infections observed in people with DS does not reflect the typical distribution of opportunistic pathogens seen in CID. In general, a large part, although not all, of the increased frequency of infection-related hospitalizations may be due to non-immunological risk factors such as anatomical reasons and their consequences (7). Moreover, for instance, despite the increase frequency of celiac disease in children with DS, we are not aware of a single case of duodenal lymphoma (32).

In summary, the observed clinical and biological abnormalities of the immune system in DS on the one hand, and the reported cancer frequency and unique distribution of malignant disease types on the other hand, suggest that immune surveillance plays little role, if any, in this context (Figure 1).

## Other Genetic Conditions Without Effective Cancer Immune Surveillance

Interestingly, trisomy 18, or Edwards syndrome (ES), is also associated with a unique tumor profile. Children with ES have an increased incidence of hepatoblastoma and nephroblastoma compared to children with a normal constitutional karyotype (33). However, extensive review of the literature indicates that hematopoietic malignancies and brain tumors, the two most frequent malignancies in children, are unusually rare in children with ES. Further, similar to DS, the immune system in fetuses with ES shows immunological defects, with a decrease of some B lymphocyte and T lymphocyte subpopulations (34, 35).

In DS and ES, the impaired immune system cannot explain the lower cancer burden and cancer incidence variations because, following the immune surveillance theory, one might expect a globally increased cancer burden. Similar to conditions with primary immune deficiency—such as common variable immune deficiency, X-linked agammaglobulinemia, selective IgA deficiency, X-linked hyper-IgM syndrome, Wiskott Aldrich syndrome, and severe congenital neutropenia (5, 6)—there is no uniform increase in all malignancies, but rather overrepresentation of a narrow spectrum of cancers, including, e.g., lymphomas, digestive tract tumors, and virus-induced tumors. Additionally, primary immunodeficiency diseases have a decreased incidence of some cancers, such as breast, lung, and colon carcinomas (36). A unique general mechanism therefore is unlikely to explain the tumor profiles of these various primary immunodeficiency disorders.

This evidence raises two important questions that largely extend beyond people with DS. First, what is the basic role of the immune system in cancer in non-therapeutic conditions? Given the increasing success of various immunotherapies in modern oncological treatment (37), it is surprising that the frequency and spectrum of malignancies in individuals with primary immune deficiencies does not reflect the corresponding mechanisms of impaired immune surveillance (6). Second, which mechanism(s) protect(s) people with DS so efficiently against the most frequent human solid tumors, particularly carcinomas? Does the presence of a third chromosome 21 offer tumor-suppressive factors?

## What is the Relationship Between Cancer And the Immune System in DS?

Considering cancer immune surveillance, primary immune deficiencies do not exhibit an important excess of all types of cancers, but rather a slight global increase due to a high frequency of lymphomas and digestive tract or virus-related cancers (36, 38). Lymphomas mostly occur in conditions with cells (lymphocyte precursors) more vulnerable toward transformation due to impaired cell maturation, function, or signaling. Digestive tract or virus-related cancers may be a consequence of microorganism infections and chronic inflammation, potentially facilitated by immunodeficiency and a lack of immune surveillance (extrinsic mechanisms). Although epigenetic and environmental factors such as a different exposure to tobacco of individuals with DS as compared to the general population may play a role, and, similarly, a different diet, intestinal microbiome, or other factors cannot be ruled out, these conditions, for which cancer incidence is based on strong epidemiological data and where the immune function is well documented, challenge the idea of a global immune-mediated protection against cancer. However, additional studies are needed to examine the model of immune surveillance in other conditions and particularly in the general population. These results do not contradict the current therapeutic successes of immune treatment in several cancers (37, 39).

## What Protects Individuals With DS From Cancers?

The broader population of people with intellectual disabilities develops a similar frequency of cancers as the general population (10–12), suggesting that the protection of individuals with DS against cancer must be linked to specific excess of genetic material on the supernumerary chromosome 21 (comprising nearly 300 genes). However, not only aberrantly expressed genes of chromosome 21 that include oncogenes and tumor suppressors, but rather complex interactions between them with genes mapping to other chromosomes lead to modified phenotypes and functions in various tissues and biological processes. Despite increased cancer risk factors—such as being overweight, low physical activity, nulliparity (for breast cancers in women) (28), and accelerated aging—sensitivity of tissues to genotoxic stress, increased DNA damage, and deficient DNA repair (40), many organs and tissues of people with DS are protected against malignant transformation, particularly breast and neural cells (but not glial cells). Thus, the “physiological” state of tissues with trisomy 21 is the result of a modified regulation of many interacting pathways that lead to tumor-protective protection. Analyzing the “interactome” (the signaling pathway-specific transcriptome and proteome) of DS tissues and comparing the exome of cancers in DS with normal DS tissues might therefore represent a possibly more fruitful approach than focusing on the effects of single genes on various functions. Because the observed profile of malignancies is not simply explained by impaired immunosurveillance, other avenues to understand reduced cancer incidence deserve additional attention. For instance, metabolic modifications in relation to the Warburg hypothesis could be considered a key context for reduced cancers in DS (41). Yet, metabolic effects on cancer occurrence have not been studied despite well-documented mitochondrial anomalies in DS (42). Other studies should more fully consider the roles of angiogenesis and stem cell availability (40).

## Conclusion

The incidence, distribution and clinical course of cancers in children and adults with DS in context with their increased risk of infections and abnormalities in the immune system do not support a model of enhanced immune surveillance providing protection from tumors. Rather, they suggest that other inherent, trisomy 21-linked, mechanisms account for the natural and strong protection against many cancer types, except leukemia and testicular cancer, in this condition. DS therefore offers an interesting condition in which to study how organisms may efficiently be protected against certain malignancies.

## Author Contributions

All authors listed have made a substantial, direct and intellectual contribution to the work, and approved it for publication.

### Conflict of Interest Statement

The authors declare that the research was conducted in the absence of any commercial or financial relationships that could be construed as a potential conflict of interest.

## References

[1] Burnet FM. The concept of immunological surveillance. Prog Exp Tumor Res. (1970) 13:1–27. 10.1159/000386035 4921480

[2] Dunn GP Bruce AT Ikeda H Old LJ Schreiber RD. Cancer immunoediting: from immunosurveillance to tumor escape. Nat Immunol. (2002) 3:991–8. 10.1038/ni1102-991 12407406

[3] Swann JB Smyth MJ. Immune surveillance of tumors. J Clin Invest. (2007) 117:1137–46. 10.1172/JCI31405 17476343 PMC1857231

[4] Finn OJ Immuno-oncology: understanding the function and dysfunction of the immune system in cancer. Ann Oncol. (2012) 23(Suppl. 8):viii 6–9. 10.1093/annonc/mds256 PMC4085883 22918931

[5] Hauck F Voss R Urban C Seidel MG. Intrinsic and extrinsic causes of malignancies in patients with primary immunodeficiency disorders. J Allergy Clin Immunol. (2018) 141:59–68.e4. 10.1016/j.jaci.2017.06.009 28669558

[6] Satgé D. A tumor profile in primary immune deficiencies challenges the cancer immune surveillance concept. Front Immunol. (2018) 9:1149. 10.3389/fimmu.2018.01149 29881389 PMC5976747

[7] Ram G Chinen J. Infections and immunodeficiency in Down syndrome. Clin Exp Immunol. (2011) 164:9–16. 10.1111/j.1365-2249.2011.04335.x 21352207 PMC3074212

[8] Satgé D Sommelet D Geneix A Nishi M Malet P Vekemans MJ. A tumor profile in Down syndrome. Am J Med Genet. (1998) 78:207–16. 9677053

[9] Coppus AM. People with intellectual disability: what do we know about adulthood and life expectancy? Dev Disabil Res Rev. (2013) 18:6–16. 10.1002/ddrr.1123 23949824

[10] Patja K Pukkala E Sund R Iivanainen M Kaski M. Cancer incidence of persons with Down syndrome in Finland: a population-based study. Int J Cancer (2006) 118:1769–72. 10.1002/ijc.21518 16231334

[11] Hasle H Friedman JM Olsen JH Rasmussen SA. Low risk of solid tumors in persons with Down syndrome. Genet Med. (2016) 18:1151–7. 10.1038/gim.2016.23 27031084

[12] Sullivan SG Hussain R Glasson EJ Bittles AH. The profile and incidence of cancer in Down syndrome. J Intellect Disabil Res. (2007) 51:228–31. 10.1111/j.1365-2788.2006.00862.x 17300418

[13] Satgé D Sasco AJ Lacour B. Are solid tumours different in children with Down's syndrome? Int J Cancer (2003) 106:297–8. 10.1002/ijc.11212 12800210

[14] Satgé D Sasco AJ Carlsen NLT Stiller CA Rubie H Hero B et al. A lack of neuroblastoma in Down syndrome: a study from 11 European countries. Cancer Res. (1998) 58:448–452. 9458088

[15] Satgé D Stiller CA Rutkowski S von Bueren AO Lacour B Sommelet D. A very rare cancer in Down syndrome: medulloblastoma. Epidemiological data from 13 countries. J Neurooncol. (2013) 112:107–14. 10.1007/s11060-012-1041-y 23307327

[16] Hermon C Alberman E Beral V Swerdlow AJ. Mortality and cancer incidence in persons with Down's syndrome, their parents and siblings. Ann Hum Genet. (2001) 65:167–76. 10.1046/j.1469-1809.2001.6520167.x 11427176

[17] Thilaganathan B Tsakonas D Nicolaides K. Abnormal fetal immunological development in Down's syndrome. Br J Obstet Gynaecol. (1993) 100:60–2. 10.1111/j.1471-0528.1993.tb12952.x 8427840

[18] Arkwright P McDermott L Immune function, infection and autoimmunity. In: Newton RW, Puri S, Marder L, editors. Down Syndrome Current Perspectives. London: Mac Keith Press (2015). p. 88–97.

[19] Arron JR Winslow MM Polleri A Chang CP Wu H Gao X et al. NFAT dysregulation by increased dosage of DSCR1 and DYRK1A on chromosome 21. Nature (2006) 441:595–600. 10.1038/nature04678 16554754

[20] Hill DA Gridley G Cnattingius S Mellemkjaer L Linet M Adami HO et al. Mortality and cancer incidence among individuals with Down syndrome. Arch Intern Med. (2003) 163:705–11. 10.1001/archinte.163.6.705 12639204

[21] Schoch J Rohrer TR Kaestner M Abdul-Khaliq H Gortner L Sester U. Quantitative, phenotypical, and functional characterization of cellular immunity in children and adolescents with Down syndrome. J Infect Dis. (2017) 215:1619–28. 10.1093/infdis/jix168 28379413

[22] Kusters MA Verstegen RH Gemen EF de Vries E. Intrinsic defect of the immune system in children with Down syndrome: a review. Clin Exp Immunol. (2009) 156:189–93. 10.1111/j.1365-2249.2009.03890.x 19250275 PMC2759463

[23] Verstegen RHJ Driessen GJ Bartol SJW van Noesel CJM Boon L van der Burg M et al. Defective B-cell memory in patients with Down syndrome. J Allergy Clin Immunol. (2014) 134:1346–53.e9. 10.1016/j.jaci.2014.07.015 25159464

[24] Martínez E Castañeda D Jaramillo S Iregui A Quiñonez T Rodríguez JA et al. Altered immune parameters correlate with infection-related hospitalizations in children with Down syndrome. Hum Immunol. (2016) 77:594–9. 10.1016/j.humimm.2016.05.004 27166175

[25] Cossarizza A Ortolani C Forti E Montagnani G Paganelli R Zannotti M. Age-related expansion of functionally inefficient cells with markers of natural killer activity in Down's syndrome. Blood (1991) 77:1263–70. 1825795

[26] Yamato F Takaya J Yasuhara A Teraguchi M Ikemoto Y Kaneko K. Elevated intracellular calcium in neutrophils in patients with Down syndrome. Pediatr Int. (2009) 51:474–7. 10.1111/j.1442-200X.2008.02761.x 19400826

[27] Alsubie HS Rosen D. The evaluation and management of respiratory disease in children with Down syndrome (DS). Paediatr Respir Rev. (2018) 26:49–54. 10.1016/j.prrv.2017.07.003 29033214

[28] Patja K Eero P Iivanainen M. Cancer incidence among people with intellectual disability. J Intellect Disabil Res. (2001) 45:300–7. 10.1046/j.1365-2788.2001.00322.x 11489051

[29] Sullivan SG Hussain R Threlfall T Bittles AH. The incidence of cancer in people with intellectual disabilities. Cancer Causes Control (2004) 15:1021–5. 10.1007/s10552-004-1256-0 15801486

[30] Gamis AS Smith FO. Transient myeloproliferative disorder in children with Down syndrome: clarity to this enigmatic disorder. Br J Haematol. (2012) 159:277–87. 10.1111/bjh.12041 22966823

[31] Pulvirenti F Pecoraro A Cinetto F Milito C Valente M Santangeli E. Gastric cancer is the leading cause of death in Italian adult patients with common variable immunodeficiency. Front Immunol. (2018) 9:2546. 10.3389/fimmu.2018.02546 30455695 PMC6230622

[32] Satgé D Sasco AJ Réthoré M-O Any Digestive Tract Tumor in Down Syndrome? About a Theoretical Risk and Practical Observation. Pediatrics P3Rs November (2006) (Letter).

[33] Satgé D Nishi M Sirvent N Vekemans M. A tumor profile in Edwards syndrome (trisomy 18). Am J Med Genet C Semin Med Genet. (2016) 172:296–306. 10.1002/ajmg.c.31511 27474103

[34] Makrydimas G Plachouras N Thilaganathan B Nicolaides KH. Abnormal immunological development in fetuses with trisomy 18. Prenat Diagn. (1994) 14:239–41. 10.1002/pd.1970140403 8066033

[35] Zizka Z Calda P Fait T Haakova L Kvasnicka J Viskova H. Prenatally diagnosable differences in the cellular immunity of fetuses with Down's and Edwards' syndrome. Fetal Diagn Ther. (2006) 21:510–4. 10.1159/000095663 16969005

[36] Mayor PC Eng KH Singel KL Abrams SI Odunsi K Moysich KB. Cancer in primary immunodeficiency diseases: cancer incidence in the United States immune deficiency network registry. J Allergy Clin Immunol. (2017) 141:1028–35. 10.1016/j.jaci.2017.05.024 28606585 PMC5723251

[37] Maus MV Grupp SA Porter DL June CH. Antibody-modified T cells: CARs take the front seat for hematologic malignancies. Blood (2014) 123:2625–35. 10.1182/blood-2013-11-492231 24578504 PMC3999751

[38] Vajdic CM Mao L van Leeuwen MT Kirkpatrick P Grulich AE Riminton S. Are antibody deficiency disorders associated with a narrower range of cancers than other forms of immunodeficiency? Blood (2010) 116:1228–34. 10.1182/blood-2010-03-272351 20466855

[39] Larkin J Chiarion-Sileni V Gonzalez R Grob JJ Cowey CL Lao CD Combined nivolumab and ipilimumab or monotherapy in untreated melanoma. N Engl J Med. (2015) 373:23–34. 10.1056/NEJMoa1504030 26027431 PMC5698905

[40] NiŽetić D Groet J. Tumorigenesis in Down's syndrome: big lessons from a small chromosome. Nat Rev Cancer (2012) 12:721–32. 10.1038/nrc3355 22996602

[41] Warburg O. On the origin of cancer cells. Science (1956) 123:309–14. 10.1126/science.123.3191.309 13298683

[42] Valenti D Braidy N De Rasmo D Signorile A Rossi L Atanasov AG. Mitochondria as pharmacological targets in Down syndrome. Free Radic Biol Med. (2018) 114:69–83. 10.1016/j.freeradbiomed.2017.08.014 28838841

